# Evaluating the relationship between time‐domain indices of ultra‐short term heart rate variability and cognition: Findings from the Singapore Longitudinal Ageing Study

**DOI:** 10.1002/alz70856_106586

**Published:** 2026-01-09

**Authors:** Tony Chin Ian Tay, Iris Rawtaer, Shiou Liang Wee, Tze Pin Ng, Caroline Sévoz‐Couche

**Affiliations:** ^1^ Singapore Institute of Technology, Singapore, Singapore; ^2^ Sengkang General Hospital, Singapore, Singapore, Singapore; ^3^ Singapore University of Social Sciences, Singapore, Singapore, Singapore; ^4^ Khoo Teck Puat Hospital, Singapore, Singapore, Singapore; ^5^ Centre de recherche en Epidémiologie et Santé des Populations, Paris, Orsay, France

## Abstract

**Background:**

The link between cardiovascular health and cognitive impairment has been documented in the literature. Heart rate variability has the potential to be a biomarker of cognitive impairment as it reflects the sympathetic and parasympathetic cardiac control mechanism of the nervous system. This study investigates the association between ultra‐short term heart rate variability and cognitive measures among older adults using electrocardiogram obtained from the Singapore Longitudinal Ageing Study.

**Method:**

A retrospective study where ultra‐short term heart rate variability was calculated through electrocardiogram obtained from the second cohort of the Singapore Longitudinal Ageing Study. 140 participants above the age of 60 years old were selected from the baseline visit. Indices of heart rate variability such as R‐R interval, SDNN, r‐MSSD, SD1, SD2/SD1 ratio and heart rate per minute were calculated. Spearman's rank‐order correlations was conducted to determine the association between time‐domain indices of ultra‐short term heart rate variability and cognitive measures.

**Result:**

There were positive and significant correlations observed between SD2/SD1 ratio and RAVLT immediate memory (*r*_s_ = .22, *n* = 140, *p* < 0.01), SD2/SD1 ratio and RAVLT delayed recall (*r*_s_ = .22, *n* = 139, *p* < 0.05), SD2/SD1 ratio and Block Design test (*r*_s_ = .19, *n* = 140, *p* < 0.05) and R‐R mean and Color Trial Test 1 (*r*_s_ = .17, *n* = 138, *p* < 0.05). Meanwhile, a significant negative correlation was found between r‐MSSD and Boston Naming Test (*r*_s_ = ‐.21, *n* = 140, *p* < 0.05).

**Conclusion:**

These results suggest an association between parasympathetic modulation and cognitive performance for measures of memory, visuospatial ability and executive function among community‐dwelling older adults. Following these results, we hope to explore the potential use of ultra‐short term heart rate variability in differentiating between older adults with mild cognitive impairment and healthy cognition.

